# Effect of valsartan on atrial fibrillation recurrence following pulmonary vein isolation in patients

**DOI:** 10.3892/etm.2014.2143

**Published:** 2014-12-16

**Authors:** YINGKAI CUI, CHANGSHENG MA, DEYONG LONG, LIPING WANG, XUEBIN CAO, GHANG ZHANG

**Affiliations:** 1 Department of Cardiology, Beijing Anzhen Hospital, Capital Medical University, Beijing Institute of Heart, Lung and Blood Vessel Diseases, Chao Yang, Beijing 100029, P.R. China; 2 Department of Cardiology, Geriatric Cardiovascular Disease Center of Chinese PLA, No. 252 Hospital of PLA, Baoding, Hebei 071000, P.R. China

**Keywords:** atrial fibrillation, pulmonary venous ablation, angiotensin receptor blocker

## Abstract

Pulmonary venous isolation has emerged as an effective therapy for atrial fibrillation (AF); however, AF recurrence is common. The aim of the present study was to investigate the effect of angiotensin receptor blockers (ARBs) on the recurrence rate of AF following ablation therapy. In total, 120 patients, who were scheduled for ablation, were randomly selected. The patients were randomly divided into three groups, which received treatment with a placebo (n=40), 80 mg valsartan daily (n=40) or with 160 mg valsartan daily (n=40). The demographic characteristics, comorbidities, AF type and information regarding treatment with ARBs were recorded and analyzed. Following a mean follow-up period of 13.8±8.6 months, 66.7% of patients were found to be free of AF. Kaplan-Meier analysis of the time until the first recurrence during the follow-up period revealed that patients treated with 160 mg/day valsartan presented a higher probability of remaining free of AF (88%, vs. 47% for the control and 65% for the 80 mg/day valsartan groups). In addition, multivariate analysis demonstrated that treatment with ARB was associated with lower AF recurrence rates (hazard ratio, 0.46; 95% confidence interval, 0.20–0.93] P=0.01). In conclusion, treatment with 160 mg/day valsartan markedly reduced the risk of recurrence of AF in a dose-dependent manner in AF patients following ablation.

## Introduction

Atrial fibrillation (AF) is the most common type of cardiac arrhythmia; however, current therapies are not efficient in the management of this condition (1). Although multiple treatment methods exist, no single modality is effective in all patients. Pulmonary vein isolation has emerged as an effective method for the treatment of AF. However, recurrences are frequent and occur in 10–40% of patients (2–4). An increasing number of clinical and experimental studies have revealed that AF is associated with atrial electrical and structural remodeling (5). Valsartan (recognized by the trade name, Diovan) is an angiotensin II (Ang II) receptor antagonist, also known as Ang II receptor blocker (ARB), which possesses a particularly high affinity for the type I Ang II (AT_1_) receptor (6). A previous study in atrial rapid-pacing dogs has demonstrated that valsartan may prevent the induction and promotion of AF through the attenuation of calpain I upregulation and the suppression of atrial structural remodeling (7). Furthermore, Ang II has been demonstrated to be involved in the mediation of inflammatory responses, which are also involved in the development of AF (8). However, previous studies on the comparative effects of the administration of Ang-converting-enzyme inhibitor (ACEI) or ARB on the incidence of AF have reported inconsistent findings (9–11). Despite some studies demonstrating that ARB may not lower the incidence of AF, further studies are required due to the multiple biological effects of ARB. The aim of the present study was to evaluate the efficacy of treatment with ARBs in patients undergoing antral pulmonary vein isolation.

## Materials and methods

### Participants

In total, 120 randomly selected patients, referred to the Arrhythmia Unit of the Beijing AnZhen Hospital (Beijing, China) for AF ablation therapy between December 2008 and July 2012, were enrolled into this study. Patients developing major postablation complications or in which AF ablation was considered to be unsuccessful (patients presenting permanent AF) were not included in the analysis, due to the low possibility of late conversion to sinus rhythm in these patients, and the effect of renin-Ang-aldosterone system (RAAS) inhibitors in decreasing AF could not be demonstrated. Further exclusion criteria included: Secondary causes of hypertension; congestive heart failure with symptoms classified as class II to IV, according to the New York Heart Association functional classification (12); left ventricular ejection fraction of <35%; previous coronary artery stenting or angioplasty; previous AF ablation procedure; transverse left atrial diameter of 60 mm, detected by transthoracic echocardiography; administration of amiodarone treatment; valvular diseases; and suffering from type 1 diabetes mellitus. This study was approved by the Medical Ethics Committee of Beijing AnZhen Hospital. All the patients provided informed written consent prior to participation in this study.

### Study design

All the patients were randomly divided, by computer-generated randomization, into the control (placebo), 80 mg/day valsartan or 160 mg/day valsartan (Novartis AG, Basel, Switzerland) groups. The variables considered in this study included: age, gender, body mass index, presence/absence of coronary artery disease, type of AF (paroxysmal and persistent), hypertension and diabetes mellitus. Baseline examinations were performed prior to ablation. The patients administered ACEIs or ARBs for the treatment of hypertension were also administered a calcium antagonist at least four weeks before AF ablation. Three weeks before ablation, all the patients were administered the anticoagulant, warfarin (target international normalized ratio, 2.0–3.0; Shanghai Xinyi Pharmaceutical Co., Ltd., Shanghai, China), unless contraindicated. Patients free from AF were further adminsitered warfarin three months after ablation. Subsequently, the patients were subjected to pulmonary vein isolation and were administered valsartan. The valsartan dose of 160 mg/day was reached by administration in two stages, lasting one week each; the patients were admininstered 80 mg/day in the first week, and 160 mg/day in the next week. For all the patients, follow-up was performed for at least one year after ablation. The initial three months after AF ablation were considered to be the ‘blanking period’, during which AF recurrence was not considered as an indicator of ablation failure. The patients were examined each month within the first 3 months, followed by examination at six and twelve months after AF ablation, as well as on any other occasions when the patients presented symptoms. A 24-h Holter recording was performed at one, six and twelve months after ablation, in order to monitor the electrocardiograms of the patients. Prior to AF ablation, all the patients were subjected to delayed enhancement cardiac magnetic resonance imaging to determine the extent of atrial fibrosis (13). In order to determine the extent of myocardial fibrosis, the patients received intravenous gadolinium (0.1 mmol/kg body weight; MultiHance^®^; Bracco Diagnostics Inc., Princeton, NJ, USA).

### Outcome measurements

The present study compared the administration of 80 mg/day valsartan, 160 mg/day valsartan and a placebo to assess the efficacy of different doses of valsartan based on the cumulative number of patients relapsing into AF following ablation to maintain the sinus rhythm. The primary endpoint was failure of the AF treatment during the follow-up. The beginning of the follow-up period for this purpose was considered to be the day of the ablation. A blanking period of three months was used after ablation, and recurrences within the first three months were not classified as failure of the ablation. Any patient suffering a persistent atrial arrhythmia during this period was reverted to sinus rhythm by electrical cardioversion.

### Statistical analysis

Statistical analysis was performed using the SPSS software (version 13.0; SPSS, Inc., Chicago, IL, USA). The results are expressed as the mean ± standard deviation. Categorical variables were compared by χ^2^ analysis, while continuous variables were compared by Student’s t-test. Kaplan-Meier analysis was performed to determine the probability of treatment success, which was expressed as the percentage of patients remaining free from AF. Differences in arrhythmia-free survival were assessed with the log-rank test. The recurrence of AF was analyzed using the Cox proportional-hazards regression, to regulate potentially confounding factors. P<0.05 was considered to indicate a statistically significant difference.

## Results

In total, 120 patients were included in the analysis. A total of 40 patients were randomly selected for treatment with a placebo, while 40 patients were treated with 80 mg/day valsartan and 40 patients were treated with 160 mg/day valsartan. The baseline clinical characteristics (prior to ablation) of each group are shown in Table I. No statistically significant differences were observed in all the pre-treatment characteristics among the three treatment groups. The median duration of AF prior to randomization was 8.6±7.2, 9.7±8.2 and 9.1±7.8 months in the 80 mg/day valsartan, 160 mg/day valsartan and placebo groups, respectively, with no statistically significant differences observed among the three groups. No patients succumbed to the disease or were not able to follow-up during the study.

Following the completion of a mean follow-up period of 12.1±9.6 months, 92 patients were found to be free from AF (Table II). At the end of the follow-up period, 21/40 patients treated with 80 mg/day valsartan, 38/40 patients treated with 160 mg/day valsartan and 18/40 patients treated with a placebo were found to be free of AF. The systolic blood pressure (SBP) and diastolic blood pressure (DBP) at the end of the follow-up period were significantly different in patients treated with 80 mg/day (SBP, 114±19 mmHg; DBP, 72±11 mmHg DBP) and 160 mg/day valsartan (SBP, 125±10 mmHg; DBP, 71±8 mmHg) valsartan, when compared with the control patients (SBP, 130±15 mmHg; DBP, 78±13 mmHg). Univariate analysis identified that P=0.3 for SBP and P=0.03 for DBP.

**Figure 1:**
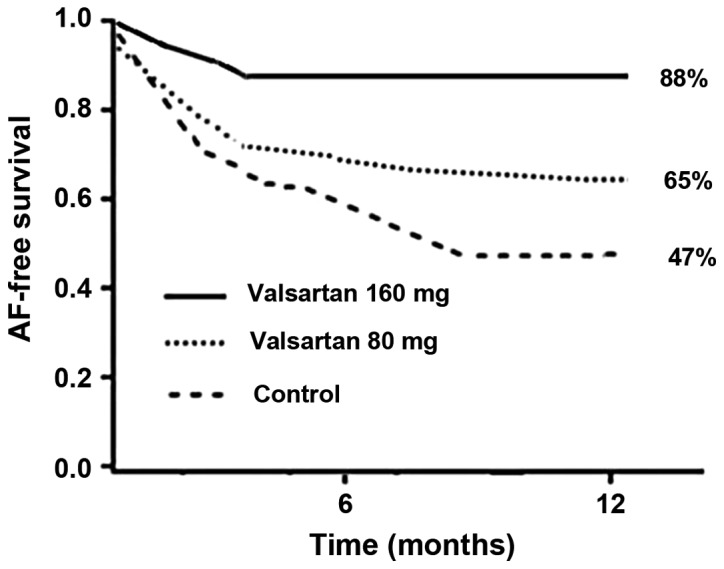
Kaplan-Meier curves of the percentage of patients remaining free from recurrence of atrial fibrillation, demonstrating the time between randomization and the first recurrence of atrial fibrillation, as determined by electrocardiography. The follow-up period (months) began following ablation.

Kaplan-Meier analysis demonstrated that the 12-week probability for maintaining sinus rhythm was 92% in patients receiving 160 mg/day valsartan, whereas the probability was 76% in patients receiving 80 mg/day valsartan and 67% in patients receiving placebo treatment (P<0.05). The Kaplan-Meier analysis of the time until the first recurrence during the follow-up period revealed that patients treated with 160 mg/day valsartan presented a greater probability of remaining free of AF (88% in the 160 mg/day valsartan group, vs. 65% in the 80 mg/day valsartan group and 47% in the placebo group; Fig. 1). Treatment with 80 mg/day valsartan was not found to decrease the recurrence rate of AF when compared with the control group (P=0.07). No statistically significant difference was observed between the 80 and 160 mg/day valsartan doses (P=0.06), whereas the difference between the 160 mg/day valsartan and control groups was statistically significant (P=0.01).

Multivariate analysis revealed that the use of 160 mg/day valsartan was the only significant variable associated with the maintenance of sinus rhythm following ablation. The Cox proportional model was used to correct for different variables (including age, gender, AF duration, left atrium size, SBP and DBP), which may influence the results. Treatment with 160 mg/day valsartan was found to be associated with a lower risk of AF recurrence (hazard ratio, 0.46; 95% confidence interval, 0.20–0.93; P=0.01).

## Discussion

A randomized trial was performed in the present study, comparing the effect of two doses of valsartan (80 and 160 mg/day) on the recurrence rate of AF following ablation in 120 patients. The results indicated that the recurrence rate of AF was affected in a dose-dependent manner and was lower in the group treated with 160 mg/day valsartan.

Atrial fibrillation (AF) is the most common sustained arrhythmia in clinical practice (14). Catheter ablation has emerged as an effective therapy for AF that does not respond to other medical treatments (15). However, catheter ablation is limited by significant recurrence rates of 10–40%, depending on various factors, including left atrial size, presence of persistent AF, scarring in the left atrium and AF duration (15–17). These variables are associated with the remodeling process, which may help trigger and maintain AF (18). Electrical and structural remodeling have been previously demonstrated to be associated with inflammation and oxidative stress (19).

The progression of atrial alteration is a fundamental component of AF pathophysiology. The RAAS is directly and indirectly involved in the development of the AF substrate (20,21). Previous animal studies have demonstrated that inhibition of RAAS may prevent AF (22,23). Furthermore, antagonists of the RAAS may have an antifibrotic effect, due to their ability to decrease the synthesis of collagen type I molecules, as well as increase the degradation of collagen type I fibres (24). A previous study has demonstrated that a blockade of the AT_1_ receptor reduces the development of atrial fibrosis and, thus, chronic structural remodelling (9). Although the precise signaling pathways resulting in atrial fibrosis remain unclear, evidence indicates that activation of the RAAS may result in atrial fibrosis. A number of studies have identified a positive interaction between ARBs and potassium channel blockers on transmembrane action potentials and currents (25,26). In addition, ARBs have been demonstrated to modify the cardiac delayed rectifiers, hKv1.5, HERG and Ks currents, indicating that ARBs may possess antiarrhythmic properties.

Valsartan is a potential drug for the treatment of hypertension, congestive heart failure or myocardial infarction (27). A study by Li *et al* demonstrated for the first time that valsartan may prevent the induction and promotion of AF, through the attenuation of calpain I upregulation and suppression of atrial structural remodeling in atrial rapid-pacing dogs (7). In addition, Harada *et al* identified that the transient receptor potential canonical type-3 (TRPC3) channel plays a critical role in AF-promoting fibroblast pathophysiology, which is a novel potential therapeutic target (28). Furthermore, valsartan may affect the fibrotic process by inhibiting TRPC3, which has been found to be significantly upregulated in the atrium of canines suffering from AF (29).

In the present study, treatment with 160 mg/day valsartan resulted in a significantly reduced AF recurrence rate, when compared with the control or 80 mg/day valsartan groups, which is in agreement with the observations of previous studies (30,31). For instance, Parving *et al* demonstrated the dose-dependent effect of irbesartan in the prevention of diabetic nephropathy (30). In addition, Madrid *et al* reported that the treatment combination of irbesartan and amiodarone decreased the rate of AF recurrences in lone AF patients, in a dose-dependent manner (31).

In patients suffering from paroxysmal or persistent AF, treatment with 160 mg/day valsartan following AF ablation was found to be more effective in reducing the AF recurrence rate, when compared with the administration of 80 mg/day valsartan. These results indicated that the antiarrhythmic effect of valsartan may be associated with atrial structural remodeling, as well as specific and selective electric remodeling through the improvement of atrial conduction disturbances. In conclusion, treatment with 160 mg/day valsartan markedly reduced the risk of AF recurrence in a dose-dependent manner.

## Figures and Tables

**Table I:** Main demographic, clinical and echographic characteristics of patients in the three treatment groups.

Variable	Control (n=40)	80 mg/day valsartan (n=40)	160 mg/day valsartan (n=40)
Age, years	63.4±8.5	62.5±8.1	63.7±9.2
Male gender, n (%)	18 (45.0%)	17 (42.5)	22 (55.0)
Weight, kg	75.3±14.1	74.3±12.9	78.2±15.3
SBP, mmHg	135.6±19.3	136.6±20.1	135.8±18.7
DBP, mmHg	76.3±11.2	80.1±12.8	79.6±11.6
TC, mg/dl	187.3±13.2	179.5±11.8	189.5±14.2
HDL-C, mg/dl	34.6±5.2	38.7±5.3	33.5±4.8
FPG, mg/dl	106.2±10.0	110.7±11.1	109.7±10.8
HR, beats/min	76.2±12.1	72.7±10.1	77.9±13.1
AF duration, months	8.6±7.2	9.7±8.2	9.1±7.8
Episodes of AF, n	2.6±0.7	2.4±0.6	2.7±0.9
Ejection fraction, %	61.0±8.2	60.8±8.2	60.1±7.9
Pre-ablation atrial fibrosis, n	17.2±11.8	17.8±12.0	17.3±11.8
BMI	23.6±2.4	24.1±2.5	23.8±2.4
Presence of CAD (n)	7 (17.5)	8 (20)	8 (20)
Type of AF, n (%)			
Paroxysmal	33 (82.5)	32 (80)	30 (75)
Persistent	7 (17.5)	8 (20)	10 (25)

SBS, systolic blood pressure; DBP, diastolic blood pressure; TC, total cholesterol; HDL-C, high-density lipoprotein cholesterol; FPG, fasting plasma glucose; HR, heart rate; AF, atrial fibrillation; BMI, body mass index; CAD, coronary artery disease.

**Table II:** Results of the intention-to-treat analysis.

Variable	Control (n=40)	80 mg/day valsartan (n=40)	160 mg/day valsartan (n=40)
Sinus rhythm (three months after ablation), n	26	30	38
Sinus rhythm (end of follow-up), n	18	21	36
Systolic blood pressure (end of follow-up), mmHg	125±10	130±15	114±19
Diastolic blood pressure (end of follow-up), mmHg	71±8	78±13	72±11
Mortality rate (end of follow-up), n	0	0	0
